# *Ceratonia siliqua L* improved cryodamage of asthenozoospermic specimens: An experimental study

**DOI:** 10.18502/ijrm.v20i12.12564

**Published:** 2023-01-09

**Authors:** Fatemeh Javanmard, Ali Bidmeshkipour, Javad Ghasemian Yadegari

**Affiliations:** ^1^Department of Biology, Faculty of Basic Science, Razi University, Kermanshah, Iran.; ^2^Department of Pharmacognosy, School of Pharmacy, Lorestan University of Medical Sciences, Khorramabad, Iran.

**Keywords:** Asthenozoospermia, Ceratonia, Cryopreservation, Fertility preservation, Infertility, Male.

## Abstract

**Background:**

Sperm freezing is an important procedure in assisted reproductive technology. Freezing results in physical and chemical changes in the sperm. *Ceratonia siliqua L* (*C.siliqua*) is a tree that has antioxidant properties.

**Objective:**

This study aimed to investigate the effect of different concentrations of *C.siliqua *in a freezing medium on semen parameters, and some biochemical parameters in asthenozoospermic specimens.

**Materials and Methods:**

Forty asthenozoospermic specimens (semen specimens with motility < 32%) were obtained from men aged between 20-40 yr according to the World Health Organization criteria. Each sample was divided into 6 groups: I) fresh, II) control, III) 5, IV) 10, V) 20, and VI) 30 µg/ml *C.siliqua* extract were added to a freezing medium respectively. Then sperm parameters, malondialdehyde, total antioxidant capacity, reactive oxygen species, and sperm DNA assay were evaluated using related protocols after thawing.

**Results:**

Data analysis shows that sperm parameter, and total antioxidant capacity level increased at a concentration of 20 µg/ml of *C.siliqua* extract compared to the other concentrations of *C.siliqua* extract after cryopreservation and thawing (p < 0.001). Also, the sperm DNA fragmentation assay, reactive oxygen species, and malondialdehyde levels were significantly reduced by adding 20 µg/ml of *C.siliqua* extract to the sperm freezing medium compared to the other treated groups after cryopreservation (p < 0.001).

**Conclusion:**

*C.siliqua* extract significantly improved sperm parameters after cryopreservation and thawing in asthenozoospermic specimens, and the greatest impact was observed at the 20 µg/ml *C.siliqua L* extract concentration (p < 0.001).

## 1. Introduction

Sperm cryopreservation has been used for fertility preservation before factors such as chemotherapy, radiotherapy, and surgery, certain diseases that may lead to testicular dysfunction, subfertility, or sterility due to gonad removal caused by adjuvant therapy (1). One of the achievements of assisted reproductive technology was the clinical use of cryopreservation for the preservation of spermatozoa in the past decade (2). The rapid cryopreservation process is based on the increasing and decreasing the temperature by immersion in liquid nitrogen. Rapid cryopreservation is a low cost, simple, and safe procedure for sperm cryopreservation (3). Despite the benefits of cryopreservation, this procedure may lead to deleterious changes in sperm structure and function (4).

The sperm preparation is done for cryopreservation, which removes the antioxidant source (5). Cryopreservation causes lipid peroxidation in the sperm membrane leading to the formation of reactive oxygen species (ROS) (6). ROS initiates a chain of chemical reactions called lipid peroxidation that produces malondialdehyde (MDA), which leads to the destruction of the cell membrane (7). An increase in ROS can alter the sperm antioxidant defense system, leading to DNA damage of cells (8) that induces caspase activation and apoptosis (9). Antioxidant supplements such as vitamin C, vitamin E, selenium, zinc, CoQ10 (10-12), and ellagic acid (13) have shown positive effects on sperm cryopreservation as they can improve semen parameters (sperm motility, viability, and function) in infertile men.

Therefore, studies have shown that enriching media freezing with herbal extract antioxidants can reduce the harmful effects of sperm cryopreservation (14, 15). *Ceratonia siliqua L (C.siliqua) *is an evergreen tree with a height of 12-17 m, a species of *Bacchillas*. *C.siliqua *contains high levels of fiber, minerals such as sodium, potassium, phosphorus, calcium, iron, and polyphenol, as well as vitamins such as D, C, E, B6, folic acid, and niacin (16, 17). This plant has antibacterial (18), anticancer, antidiabetic (19), and antioxidant properties due to the presence of phenolic components (20).

It has also been shown that the supplementation of freezing media with *C.siliqua* extract improved sperm progressive motility, normal morphology, viability, and sperm DNA integrity after cryopreservation and thawing in normozoospermic aged men (21). It has been reported that using *C.siliqua* fruit hydroalcoholic extract improved sperm parameters (count, motility, morphology, viability), and elevated total antioxidant capacity (TAC), antioxidant enzymes activity of serum, and decreased serum MDA levels in male mice (22).

Cryopreservation caused a significant decrease in sperm parameters (motility, normal morphology, viability, and sperm DNA integrity) compared to fresh samples. To the best of our knowledge, there are no publications on the effects of *C.siliqua* hydroalcoholic extract as an herbal antioxidant on cryopreservation in asthenozoospermic men. Therefore, in this study, the effect of *C.siliqua *hydroalcoholic extract on semen parameters, sperm DNA integrity, and some biochemical parameters after freezing in fertile men with asthenozoospermic was investigated.

## 2. Materials and Methods

Forty asthenozoospermic specimens (20-40 yr old) were obtained from men who were referred to the in vitro fertilization Unit of Shafa hospital, Lorestan, Iran, from December 2021-February 2022. The exclusion criteria were having a history of chemotherapy, recent hormonal treatment, varicocele, associated genital tract infection, alcohol intake, drug abuse, systemic diseases, smoking.

### Semen collection and preparation

This research, examined semen samples of asthenozoospermic men (20-40 yr old). According to the World Health Organization (WHO) criteria, 40 asthenozoospermic specimens (semen specimens with motility < 32%) were collected.

After liquefaction at 37 C, semen analysis was performed according to published guidelines of the WHO. According to the WHO criteria simple wash preparation was used for each asthenozoospermic specimen (23).

### Study design

Each liquefied asthenozoospermic specimen was divided into 6 groups: I) fresh (before cryopreservation), II) control (cryopreserved without *C.siliqua* extract), III) 5, IV) 10, V) 20, and VI) 30 µg/ml *C.siliqua* extract were added to a freezing medium respectively. All assessments were done before cryopreservation and after thawing. These doses were according to the previous research (15).

### Rapid cryopreservation and thawing

Rapid cryopreservation and thawing processes were applied to provide semen specimen. To dilute the specimen, an equal volume of sperm freezing solution (Sperm Freeze Solution, Vitrolife, Goteborg, Sweden) was added to each specimen in the cryovial at room temperature. Sperm freezing solutions should be added slowly, drop by drop, to the microvial containing semen so that the mixture is tilted after adding each drop. Then the cryovial lids are tightly closed and turned upside down 20 times in order to avoid any bubble formation. The semen mixtures were kept at room temperature for 10 min, and then the mixture was vaporized at above 1-3 cm of liquid nitrogen for 30 min. Finally, they were kept at -196 C for one month after initial immersion in liquid nitrogen.

For thawing, the cryovials were removed from liquid nitrogen and placed into a 35 ± 2 C water bath for 10 min. Then, the cryovials were dried with a clean paper towel. The semen mixture was transferred to clean test tubes. The equilibrated Ham's F-10 was added slowly to the semen mixture (1:1) into test tubes and centrifuged to delete the cryoprotectant. In the end, the sperm pellets were resuspended with a sufficient amount of Ham's F-10 medium for other evaluations.

### Sperm parameters assessment

Sperm count and motility were evaluated for 200 spermatozoa by light microscopic (Olympus, Tokyo, Japan) observations (magnification = 40 × ) according to the WHO guidelines criteria. For sperm count, a Neubauer hemacytometer was used and reported as 10^6^/mL of semen. The motility of sperms was reported as the percentage of progressive, nonprogressive, and immotile sperms. To evaluate the viability and sperm morphology, the eosin test and Papanicolaou staining were performed according to WHO criteria, respectively. At least 200 spermatozoa per participants were evaluated. In the eosin staining technique, red or dark pink spermatozoa were determined dead, and noncolored head spermatozoa were considered alive (23).

### Sperm DNA fragmentation assay (SDFA)

The determination of SDFA was carried out using an SDFA kit (DAIAN ZIST AZMA CO, Iran). This assay was performed according to the manufacturer's instructions. A sperm aliquot of each sperm specimen was mixed in physiological serum or phosphate buffer solution to a maximum of 20 × 10^6^ cells per ml. A sperm aliquot of each specimen (50 μl) was mixed with 50 μl low melting point agarose. Then, 50 μl of the suspension was placed on a glass slide and covered by a coverslip. The slides were put on a surface at a temperature of 28 C for 5 min. They were immersed in denaturation solutions and incubated for 7 min in the darkroom. These slides were put into a lysis solution and incubated for 15 min at room temperature. The slides were washed with distilled water. Then, slides were immersed in a gradually reduced ethanol concentration for 2 min at each concentration (in 70, 90, and 100 ethanol) for 2 min, for dehydrated, respectively. The slides*, *after staining, were washed with distilled water in the next step. Then by light microscopy (Olympus Co., Tokyo, Japan) (100x), at least 200 spermatozoa were analyzed. In spermatozoa with fragmented DNA, the head is without a halo or has a small halo, and sperms with medium and large halos were reported as intact DNA (24).

### Biochemical parameters

#### MDA

Using the ZellBio MDA assay kit (ZB-MDA-96A, ZellBio GmbH, Germany), MDA levels of seminal plasma were determined. After adding 50 μl of reagent 4 to 50 μl of semen samples in the microtubes, 1 μl of ready chromogen solution was added to the mixtures in the microtubes. The microtubes were placed in a boiling water bath for one hr. After boiling, cooled in the ice bath. These microtubes were centrifuged for 10 min (3000-4000 g), and the supernatants were placed in the microplates. The absorbance of the supernatant was read by a microplate reader/enzyme linked immunosorbent assay (ELISA) reader at 535 nm (25).

#### TAC

Zellbio TAC Assay Kit (ZB-TAC-96A, ZellBio GmbH, Germany) was used to measure TAC. In the manner of the instructions of manufacturers, after semen centrifugation (600 g for 10 min), 10 µl sampled sperm or trolox reconstituted in various condensations (as standard), and 190 µl prepared working chromogen reagent was added to each well of microplate. The plate was covered and incubated at room temperature for 2 min, and absorption was read at 460 nm by ELISA plate reader (25).

#### ROS

“ROS” defines a class of endogenous, highly reactive, unstable molecules. ROS assay kit, which uses a ROS-sensitive probe dichlorofluorescein diacetate (DCFH-DA, D6883, Sigma-Aldrich, St. Louis, MO, USA), works based on an assessment of fluorometry. When DCFH-DA is outspread in cells, the ester-hydrolyzed enzyme hydrolyzes it into a non-fluorometric compound and quickly oxidizes it to a heavily green fluorescence dichlorofluorescein (DCF). The intensity of fluorescence has a relationship with levels of ROS in cells. Briefly, 100 mM of DCFH-DA was prepared in DMSO and 100 μl of DCHF-DA solution was added to 300 μl of the semen sample. Then, samples were incubated for 40 min at 25 C in the darkness, and the fluorescence intensity as a relative fluorescence unit was repor at 485 and 520 nm by spectrometer-based ELISA reader (26).

### Preparation of *C.silique* extract

The fruits (seeds and pods) of *C.siliqua *were purchased from the market of medicinal plants, these fruit's identities were confirmed in Natural Resource Center (Herbarium No. 7123) and approved by a botanist. After cleaning and removing impurities, these fruits were ground into powder and soaked in 96% ethanol and water (50:50) for 72 hr. The solution was filtered by Whatman filter No. 40. After filtration with Whatman filter No. 40, the extract was concentrated using a rotary evaporator at 50 C. The brown extract remained and dried in an oven at 40 C. Finally, the *C.siliqua* fruit extract was stored at -20 C.

### Ethical considerations

In addition, this research was approved by the Research Ethics Committees of Kermanshah Razi University, Kermanshah, Iran (Code: IR.RAZI.REC.1400.085), and informed consent was obtained from all participants in the study.

### Statistical analysis

The current study was analyzed using SPSS version 26 (SPSS Inc., Chicago), where the significance level was set at p = 0.001. The Shapiro-Wilk and Kolmogorov-Smirnov tests were utilized for normality assessment and the one way-ANOVA followed by TOKEY POSTHOC test for quantifying the significance of differences between groups.

## 3. Results

Sperm progressive motility, normal morphology, and viability decreased in all freezed groups (II, III, IV, V, VI) compared to the fresh group (I) (p < 0.001). Supplementation of freezing media with 20 µg/ml *C.siliqua* extract (group V) significantly improved sperm progressive motility, normal morphology, and viability compared with the control group (II) and the other treated groups (III, IV, VI) (p < 0.001) (Table I). As shown in table I, the concentration of sperms was not improved after exposure to different concentrations of *C.silique* extract (p > 0.001). The amount of DNA fragmentation was found to be increased in cryopreserved groups than in fresh sperm (p < 0.001). In addition, supplementation of freezing media with 20 µg/ml *C.siliqua* extract (group V) significantly decreased DNA fragmentation compared with the control group (II) and the other treated groups (III, IV, VI) (p < 0.001) (Table I).

**Figure 1:**
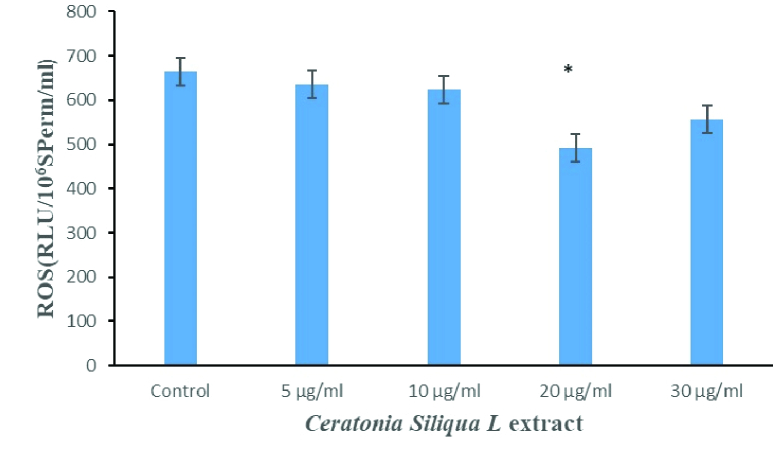
Comparison of reactive oxygen species (ROS) levels between studied groups. *P < 0.001.

The level of ROS significantly decreased in the supplementation of freezing media with 20 µg/ml * C.siliqua* extract (group V) compared with the control group and the other treated groups (Figure 1) (p < 0.001).

**Figure 2:**
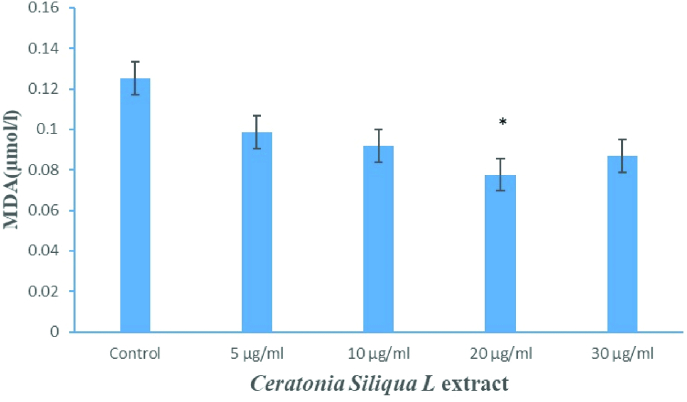
Comparison of malondialdehyde (MDA) levels between studied groups. *P < 0.001.

As shown in figure 2, MDA as an indicator of sperm lipid peroxidation significantly decreased in supplementation of freezing media with 20 µg/ml *C.siliqua *extract (group V) compared with the control group and the other treated groups (p < 0.001).

**Figure 3:**
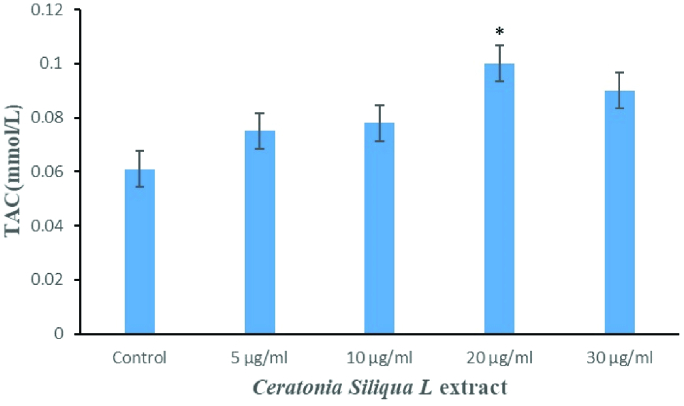
Comparison of total antioxidant levels (TAC) between studied groups. *P < 0.001.

Also, data analysis showed that the amount of TAC significantly increased in the supplementation of freezing media with 20 µg/ml *C.siliqua* extract compared to the control group and the other treated groups (Figure 3) (p < 0.001).

**Table 1:** Sperm progressive motility, concentration, normal morphology, viability, and SDFA after exposure to different concentrations of *C.siliqua* extract


**Sperm parameters**	**Fresh**	**Control**	**5 µg/ml of ** ***C.siliqua*** ** extract**	**10 µg/ml of ** ***C.siliqua*** ** extract**	**20 µg/ml of ** ***C.siliqua*** ** extract**	**30 µg/ml of ** ***C.siliqua*** ** extract**
**Concentration (×10^6^)**	41.3 ± 5.7	41.2 ± 5.7	41.0 ± 5.7	41.2 ± 5.8	41.2 ± 5.5	41.1 ± 5.8
**Progressive motility (%)**	29.37 ± 2.33	9.75 ± 2.5*	12.75 ± 2.41*#	16.42 ± 1.8*#	25.6 ± 2.34*#	20.62 ± 2.1*#
**Normal morphology (%)**	5.92 ± 1.4	2.6 ± 0.77*	2.92 ± 0.72*#	3.87 ± 0.75*#	5.07 ± 1.14*#	4.3 ± 0.99*#
**Viability (%)**	52.35 ± 10.23	29.57 ± 7.93*	33.05 ± 7.73*#	38.77 ± 8.15*#	48.47 ± 10.02*#	44.25 ± 9.08*#
**SDFA (%)**	22.33 ± 4.79	32.00 ± 4.51*	29.85 ± 4.40*#	29.17 ± 4.21*#	24.12 ± 4.87*#	27.00 ± 4.21*#
Note: Data are expressed as Mean ± SD. Wilcoxon Signed Ranks Test, Kruskal-Wallis Test, and Paired Sample Test, ANOVA. The level of statistical significance was set at p < 0.001 and was shown with* and #*Difference between group I and other groups, #Difference between group II and groups III, IV, V, VI, SDFA: Sperm DNA fragmentation assay,* C.siliqua: Ceratonia siliqua L*						

## 4. Discussion

Human sperm cryopreservation is a step in the process of fertility treatment that maintains men's fertility for years regardless of their infertility etiology. In this study, different concentrations of *C.siliqua *hydroalcoholic extracts in a freezing medium were used to optimize the sperm freezing medium.

It has been shown that it is used as herbal medicine for male infertility in several areas of Iran.

This study showed that adding different concentrations of *C.siliqua *extracts in a freezing medium significantly improved the quality of chromatin and sperm parameters in asthenozoospermic specimens. Finally, more future researches are needed to confirm the results of this study, where the findings can be used to reduce the adverse effects of freezing process. Sperm cryopreservation procedures impair sperm quality, reduce sperm fertilizing potential, and negatively affect sperm parameters (27).

Similar to previous studies (15, 22), this study revealed that the supplementation of freezing media with 20 µg/ml *C.siliqua* extract significantly improved sperm progressive motility, normal morphology, and viability compared to the other treated groups after cryopreservation in asthenozoospermic specimens.

Also, the supplementation of freezing media with 20 µg/ml of *C.siliqua* extract significantly decreased DNA fragmentation (*C.siliqua* extract improved sperm chromatin condensation) compared to the other treated groups after cryopreservation in asthenozoospermic specimens.

In the present study, the cryopreservation and thawing procedures increased ROS and MDA production. The ROS and MDA levels were also significantly reduced by adding 20 µg/ml of *C.siliqua* extract to the sperm freezing medium compared to the other treated groups after cryopreservation in asthenozoospermic specimens and reduces the adverse effects of oxidative stress and lipid peroxidation.

In addition, it has been shown that *C.siliqua* positively affects on male fertility and spermatogenesis (15). A previous study reported that *C.siliqua* extract induced by alcohol in the liver has a protective effect and causes a decrease of oxidative stress and the levels of malondialdehyde (28). In addition, many authors showed that *C.siliqua* has protective potential against oxidative stress, and it showed a significant reduction in blood glucose and biochemical profiles in diabetic rats at various doses (19).

Also, the present study demonstrated that a freezing medium supplemented with *C.siliqua *extract increased TAC levels. These results are in agreement with other studies (17, 22).

The use of the herbal extract in freezing and/or thawing media to improve sperm cryopreservation in treating male infertility has been the attention of many infertility clinics (29). In agreement with our study, it showed that adding antioxidant agents to freezing and/or thawing media might be an effective approach to improve the sperm function efficiency (30). Although, mechanisms of *C.siliqua* action on improving cryopreservation damage are not known. Moreover, it was observed that using *C.siliqua *improves cryopreservation detrimental effects on sperm properties and chromatin quality of normozoospermia in freezing/thawing situations (15).

Flavonoid and phenolic compounds (biologically active secondary plant metabolites) in the aqueous-alcoholic *C.siliqua* extract have natural antioxidant properties. It has several vitamins such as D, C, E, niacin, and folic acid, and many minerals such as calcium, iron, phosphorus, potassium, and polyphenol. It has been shown that sperm quality, biochemical parameters, and testosterone levels in infertile mice increased at a concentration of 800 mg/kg *C.siliqua* extract for 35 (17). Our results were similar to another study, which also indicated that the aqueous-alcoholic extract of *C.siliqua* fruit could improve sperm parameters, increase sex hormones, TAC, and serum antioxidant activity, and reduce serum MDA level (22).

Meanwhile, the role of articles examining medicinal herbs is quite important, and the use of herbal medicine has become more warmly embraced given that chemical medicine leaves adverse impacts on human health. Therefore, in this study, we evaluated the effect of different concentrations of *C.siliqua* extract on sperm parameters and chromatin quality after cryopreservation in asthenozoospermic specimens as an antioxidant agent to optimize sperm freezing medium. It seems that *C.siliqua* extract affects the TAC, ROS, MDA levels, and sperm DNA integrity leading to changes in the level of oxidative stress and semen parameters. *C.siliqua* extract can be used dose-dependently as a supplement in the sperm cryopreservation process which is a cost-convenient technique to help infertile men increase their fertilization rate, pregnancy rate, and ultimately, live birth. The limitation of this study is that the number of asthenozoospermic patients were low. However, we believe this is the first study to evaluate the effect of *C.siliqua* extract on sperm parameters, sperm DNA integrity, and biochemical parameters after cryopreservation in asthenozoospermic specimens.

## 5. Conclusion

This study showed that the supplementation of freezing media with *C.siliqua* extract as an antioxidant significantly improved sperm progressive motility, normal morphology, viability, and chromatin condensation after cryopreservation and thawing in asthenozoospermic specimens. Also, our results showed that the greatest impact was observed at the 20 µg/ml *C.siliqua* extract concentration in asthenozoospermic specimens (p < 0.001).

##  Conflict of Interest

No potential conflict of interest relevant to this article was reported.
